# Protein C Inhibitor—A Novel Antimicrobial Agent

**DOI:** 10.1371/journal.ppat.1000698

**Published:** 2009-12-18

**Authors:** Erik Malmström, Matthias Mörgelin, Martin Malmsten, Linda Johansson, Anna Norrby-Teglund, Oonagh Shannon, Artur Schmidtchen, Joost C. M. Meijers, Heiko Herwald

**Affiliations:** 1 Department of Clinical Sciences, Section for Clinical and Experimental Infection Medicine, Lund University, Lund, Sweden; 2 Department of Pharmacy, Uppsala University, Uppsala, Sweden; 3 Karolinska Institutet, Center for Infectious Medicine, Huddinge University Hospital, Stockholm, Sweden; 4 Division of Dermatology and Venereology, Department of Clinical Sciences, Lund University, Lund, Sweden; 5 Departments of Vascular and Experimental Vascular Medicine, Academic Medical Center, University of Amsterdam, Amsterdam, The Netherlands; Stanford University, United States of America

## Abstract

Protein C inhibitor (PCI) is a heparin-binding serine proteinase inhibitor belonging to the family of serpin proteins. Here we describe that PCI exerts broad antimicrobial activity against bacterial pathogens. This ability is mediated by the interaction of PCI with lipid membranes, which subsequently leads to their permeabilization. As shown by negative staining electron microscopy, treatment of *Escherichia coli* or *Streptococcus pyogenes* bacteria with PCI triggers membrane disruption followed by the efflux of bacterial cytosolic contents and bacterial killing. The antimicrobial activity of PCI is located to the heparin-binding site of the protein and a peptide spanning this region was found to mimic the antimicrobial activity of PCI, without causing lysis or membrane destruction of eukaryotic cells. Finally, we show that platelets can assemble PCI on their surface upon activation. As platelets are recruited to the site of a bacterial infection, these results may explain our finding that PCI levels are increased in tissue biopsies from patients suffering from necrotizing fasciitis caused by *S. pyogenes*. Taken together, our data describe a new function for PCI in innate immunity.

## Author Summary

The innate immune system is an integral part of our battle against an invading pathogen. Antimicrobial peptides and proteins partake in this fight due to their ability to perforate the bacterial cell wall, which eventually will cause the efflux of bacterial cytosolic content and efficient bacterial killing. Protein C inhibitor (PCI) is a multifunctional heparin-binding serpin which has been implicated in a number of pathological conditions, including severe infectious diseases. Here we show that PCI is a potent antimicrobial agent that is able to destroy the bacterial cell wall and thereby cause death of the bacteria. Our study also shows that in contrast to many other antimicrobial peptides, processing of PCI is not required since the full length protein exerts its antimicrobial activity, and we present data demonstrating that PCI is enriched at the infected site of patients suffering from severe streptococcal infection.

## Introduction

Our early response to an invading pathogen relies to a major part on our innate immune system. In order to sense and fight an infection, the human host has developed an arsenal of pattern recognition proteins that interact with so-called pathogen associated molecular patterns or PAMPs (for a review see [1]). Pattern recognition proteins have two major tasks. Some, like toll-like receptors, evoke an inflammatory response, such as the induction of proinflammatory cytokines [2], while others are involved in the direct killing of the pathogen. For instance, there are scavenger receptors that can act as phagocytic receptors mediating direct non-opsonic uptake of pathogenic microbes and/or their products [3]. However, there are also pattern recognition proteins, such as complement, and antimicrobial peptides that fall into both categories. For example, the anaphylatoxin peptide C3a is a potent chemoattractant for phagocytes, but also has a direct antimicrobial effect [4]; other examples include chemotactic chemokines and neuropeptides [5],[6]. The mode of the antimicrobial action of these substances is often based on their ability to penetrate the cell wall of the pathogen, which eventually leads to membrane disruption followed by cytosolic leakage and ultimately to the death of the targeted organism.

The number of antimicrobial peptides/proteins (AMPs) is constantly increasing and today more than 880 have been described [7]. In order to display their activity, many AMPs must first be released from their precursor molecules. Probably one of the best-studied mechanisms is release of LL-37 from cathelicidin hCAP-18 by the action of proteinase 3 [8]. Notably, in some cases an entire protein can exploit its antimicrobial activity without any prior processing. Thus, proteins such as bactericidal/permeability increasing protein, azurocidin and histidine-rich glycoprotein have been reported to function as antimicrobial agents (for reviews see [9],[10],[11]). It is noteworthy that many of these proteins have an affinity for heparin.

Protein C inhibitor (PCI) is a heparin-binding serine proteinase inhibitor [12]. As indicated by its name, PCI was originally reported as an inhibitor of activated protein C, a blood coagulation factor. Later it was reported that PCI is also found, apart from plasma, in tears, saliva, cerebral spinal fluid, breast milk, seminal plasma, and amniotic fluid (for a review see [13]). Recently, it was described that human PCI is efficiently internalized by neutrophils and targeted to the nucleus [14]. Interestingly, the authors also found that internalized PCI promotes phagocytosis of bacteria. As PCI apparently has an affinity for lipids, we set about to analyze its interaction with bacterial membranes. To this end we performed a number of experiments demonstrating for the first time that human PCI is a potent antibacterial reagent.

## Results

### Antimicrobial effect of SEK20

**Figure 1:**
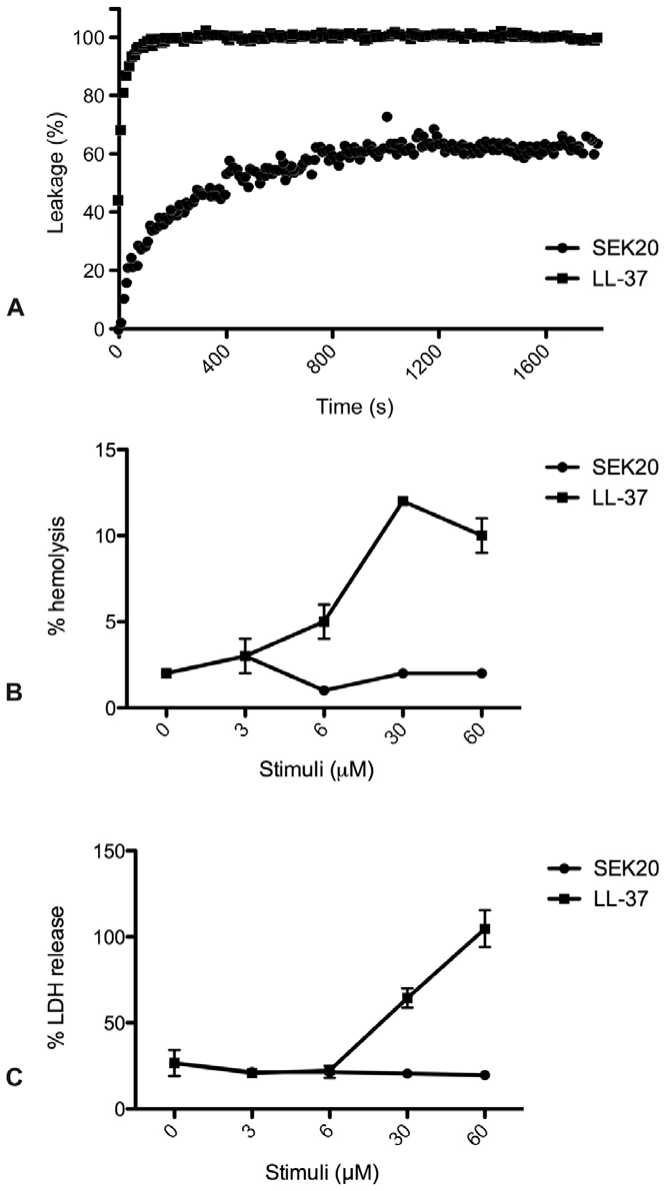
Effect of SEK20 on liposomes, HaCaT keratinocytes, and whole blood. (A) The effect of SEK20 and LL-37 on liposome permeabilization was measured in a time-dependent manner. (B) The hemolytic activity of SEK20 and LL-37 was monitored by incubating various concentrations of the peptides with human blood followed by measuring the absorbance at 540 nm. Results are expressed as % of Triton X-100 induced hemolysis. (C) Serial dilutions of SEK20 and LL-37 were added to HaCaT keratinocytes and cell permeabilization was measured by determining the release of LDH. All experiments were performed in triplicates.

Previous work has shown that SEK20, a peptide derived from PCI (SEKTLRKWLK MFKKRQLELY), and LL-37 (LLGDFFRKSK EKIGKEFKRI VQRIKDFLRN LVPRTES), have a broad antimicrobial activity against pathogens such as *Candida albicans*, *Enterococcus faecalis*, *Escherichia coli*, *Proteus mirabilis*, and *Pseudomonas aeruginosa* [15]. In addition to these pathogens we find in the present study that SEK20 is also able to kill *Bacillus subtilis*, *Staphylococcus aureus*, and *Streptococcus pyogenes*. In concordance with the previous report, the antimicrobial activity of SEK20 was as efficient as that of LL-37 (Table 1). Figure S1 shows the effect of SEK20, LL-37, and GDK25 (a control peptide derived from human high molecular weight kininogen [16]) on *E. coli* and *S. pyogenes* bacteria which were used throughout this study. Both pathogens are frequently isolated from patients suffering from severe acute infectious diseases. The broad antimicrobial activity of SEK20 and its positive net-charge (pI = 10.3) [15], suggest that the peptide does not interact with species-specific surface proteins of these pathogens, but rather targets their cell membranes, which is also the point of attack for many other antimicrobial peptides (for a review see [17]). We therefore tested the effect of SEK20 in a permeabilization assay by employing unilamellar anionic liposomes [18]. To this end, liposomes were treated with SEK20 and LL-37 and the subsequent release of carboxyfluorescein was monitored. Figure 1A shows that SEK20 like LL-37 permeabilizes liposomes, suggesting that SEK20 has membrane lytic activity.

**Table 1:** Microbial growth inhibition by LL-37 and SEK20.

Bacteria	LL-37	SEK20
*E. coli*	9.1	9.9
*P. aeruginosa*	7.8	9.0
*S. aureus*	8.3	8.2
*C. albicans*	8.2	8.5
*B. subtilis*	12.9	12.6

Microbial growth inhibition (expressed in mm) was measured using the radial diffusion assay (see also Materials and Methods). Clearance zones with a diameter more than 4 mm are considered as bacterial killing (n = 3; standard deviation was regularly less then 10%).

A negative side effect of some antimicrobial peptides is that they not only act on bacterial or fungal surfaces, but also lyse and ultimately kill eukaryotic cells. We therefore tested the effect of SEK20 on human erythrocytes and found that SEK20, in contrast to LL-37, had no hemolytic activity (Figure 1B). Similar results were obtained when measuring the LDH release from HaCaT keratinocytes, where LL-37 demonstrated significant release at higher concentrations, but SEK20 did not (Figure 1C). Taken together, the results show that SEK20 is a potent antimicrobial peptide with a broad specificity, but less toxicity for eukaryotic cells than LL-37.

### Antimicrobial activity of PCI

**Figure 2:**
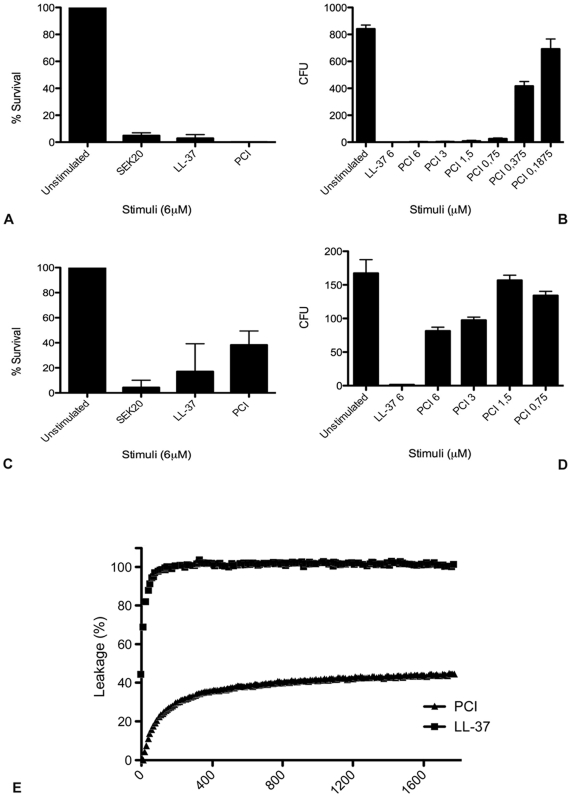
Antimicrobial effect of PCI on *E. coli and S. pyogenes*. *E. coli* 37.4 (A) and *S. pyogenes* strain AP1 (C) were incubated with SEK20 (6 µM), LL-37 (6 µM), and PCI (6 µM) or left untreated. A dose dependent effect of PCI (concentration range 0.2 - 6.0 µM) on *E. coli* 37.4 and AP1 is shown in (B and D), respectively. To quantify bactericidal activity reaction mixtures were plated on TH broth agar followed by incubation over night at 37°C and the number of cfus was determined. Statistical significance was determined using one sample T test with a theoretical mean set as 100% survival based on untreated samples. ***  = P<0.001, **  = P<0.01. Values are means, vertical lines show SEM; n = 3−5. (E) The effect of LL-37, and PCI on liposome permeabilization was measured in a time-dependent manner.

In order to become active, many antimicrobial peptides such as LL-37 must be released from a precursor molecule [19]. In some cases, however, this processing is not required and the entire protein is antimicrobial by itself, most likely because the antimicrobial region is surface exposed as is the case for histidine-rich glycoprotein and azurocidin [20],[21]. The three-dimensional structure of PCI has been resolved and a closer examination revealed that a region spanning the SEK20 sequence forms a hairpin loop sticking out at the amino terminal part of the protein [22]. It was therefore tempting to speculate that PCI is by itself antibacterial. To test this, the effect of PCI on Gram-negative (*E. coli*) and Gram-positive (*S. pyogenes*) bacteria was investigated in viable count assays. Figure 2A shows that PCI kills *E. coli* bacteria very efficiently, while its antimicrobial activity towards *S. pyogenes* bacteria is slightly reduced when compared with SEK20 or LL-37 (Figure 2C). Notably, the antimicrobial effect of PCI was dose-dependent in both cases (Figure 2B and 2D). Next, we treated PCI with several proteinases (activated human protein C, factor Xa, plasma kallikrein, thrombin, elastase, cathepsin G, and proteinase 3) in order to exclude the possibility that the activity of PCI was achieved upon proteolytic processing of the protein and subsequent release of a SEK20-containing peptide. To this end, Western blot experiments with antibodies against PCI and SEK20 revealed that the protein is resistant to proteolytic degradation and a fragment spanning the SEK20 peptide has not been released when incubated with these enzymes (data not shown). Additional analysis by negative staining electron microscopy showed that protease treatment did not affect PCI's antimicrobial activity (Figure S2). These findings are in line with reports showing that also other antimicrobial proteins such as azurocidin are resistant to proteolysis [23]. Finally, we performed binding assays with radiolabeled PCI in order to investigate the interaction between the entire PCI molecule and the bacterial surface. We found that the two bacteria strains tested, *E. coli* (12% binding of added radio-labeled protein) and *S. pyogenes* (27% binding of added radio-labeled protein), were able to assemble PCI on their surface. When ^125^I-PCI bound to streptococci was eluted from the bacterial surface and run on SDS-PAGE followed by auto-radiographic analysis, we did not find any signs of degradation (data not shown), implying that the interaction of PCI with bacteria does not trigger truncation or processing of the protein. Taken together our findings show that the entire PCI molecule has antimicrobial activity and no cleavage of the protein is needed to generate this effect.

**Figure 3:**
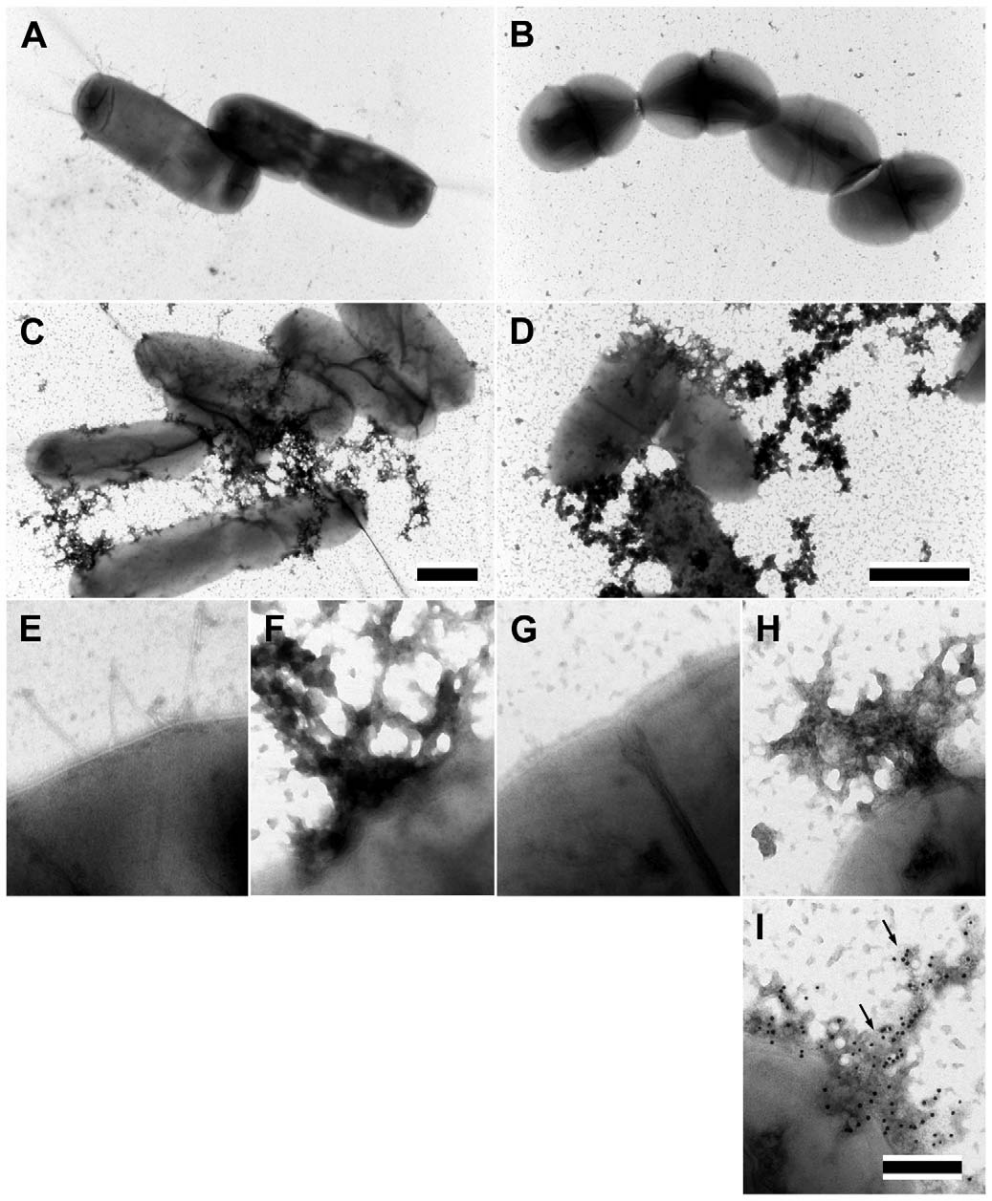
Electron microscopic analysis of *E. coli* and *S. pyogenes* bacteria treated with PCI. *E. coli* (A and C) and AP1 (B and D) bacteria before (A and B) and after (C and D) treatment with PCI 4 µM were subjected to negative staining electron microscopy (bar = 1 µm). At higher magnification, extensive membrane disruption and extravasations of cytoplasmic components is seen in PCI-treated *E. coli* (F) and AP1 (H) bacteria. Untreated *E. coli* (E) and AP1 (G) bacteria served as control (bar = 200 nm). (I) AP1 bacteria were incubated with PCI followed by incubation with a gold-labeled reversed primer coding for M1 protein (arrows), one of the most abundant proteins on the surface of AP1 bacteria (bar = 200 nm).

In the next series of experiments we wished to test whether PCI is able to permeabilize bacteria by the same mechanism as SEK20. Thus, liposomes were incubated with PCI and the release of carboxyfluorescein was recorded. The results show that PCI, like SEK20, has the ability cause a concentration dependent release of carboxyfluorescein (Figure 2E). To investigate the effect of PCI on the cell wall of *E. coli* and *S. pyogenes*, bacteria were treated with PCI and analyzed by negative staining microscopy. As seen in Figure 3, this treatment evoked significant membrane destruction, which was followed by the extravasation of cytosolic content as detected by the release of oligonucleotides (Figure 3I). An antimicrobial effect was also seen when PCI was used at 100 nM which reflects its concentration in human plasma (Figure S3). Thus, our results show that PCI mediates its antimicrobial activity by perforating the bacterial cell membrane followed by the efflux of intracellular material and subsequent death of the bacteria.

### PCI has antimicrobial activity in a plasma environment

**Figure 4:**
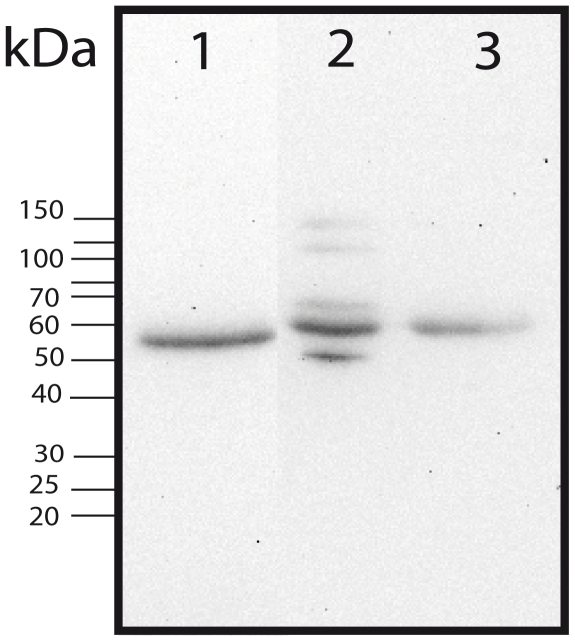
PCI is absorbed from human plasma by AP1 bacteria. *S. pyogenes* bacteria were incubated with citrated human plasma for 1 h. The bacterial cells were collected, washed with PBS, and bound proteins were eluted with 0.1 M glycine–HCl, pH 2.0. The eluted proteins were precipitated and dissolved in SDS sample buffer and subjected to Tricine-SDS gel electrophoresis followed by Western blot analysis and immunodetection with antibodies against PCI. (1) Human plasma (diluted 1∶100), (2) bacteria-bound plasma proteins and (3) human plasma (1∶100) after removal of bacteria and bacteria-bound plasma proteins.

**Figure 5:**
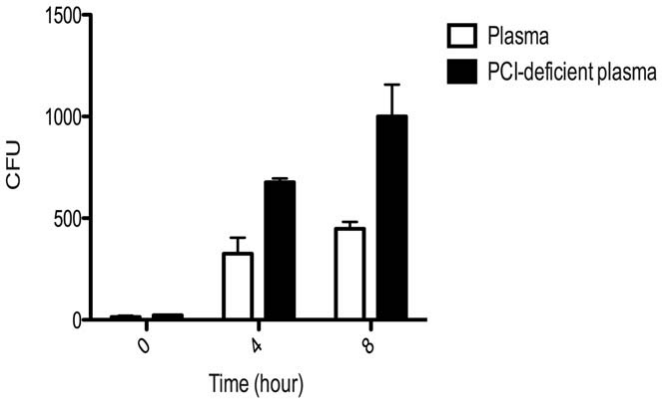
PCI inhibits bacterial growth in plasma. *S. pyogenes* strain AP1 was grown in normal human plasma or PCI-deficient plasma at 37°C for 8 h. Bacterial growth was then followed by plating appropriate dilutions of the bacterial solutions on TH agar plates at the indicated time points. Plates were incubated at 37°C over night and cfus were determined. A representative experiment of 5 is shown.

In humans, PCI is found in many fluids and secretions including plasma, seminal plasma, urine, sweat, saliva, tears, milk, and cerebrospinal fluid (for a review see [24]). Many antimicrobial peptides/proteins do not display their full activity in a physiological environment and require special conditions such as low salt concentration or low pH. To investigate whether this also applies for PCI, we studied the interaction of PCI with AP1 bacteria in human plasma. In contrast to *E. coli*, *S. pyogenes* bacteria are not phagocytozed in human blood and therefore we focused on the Streptococci only throughout the rest of this study. In a first series of experiments, we tested whether AP1 bacteria can absorb PCI from human plasma. To this end, bacteria and normal human plasma were incubated for 1 h at room temperature. After a centrifugation step to separate plasma and bacteria and a washing step, bacteria-bound plasma proteins were recovered by an acid wash and subjected to Western blot analysis with anti PCI antibodies. Figure 4 shows that PCI was recruited onto the surface of AP1 bacteria and a depletion of the protein from human plasma was also recorded. Based on these findings we wanted to explore whether PCI can exert its antimicrobial activity in plasma. In order to do so, we compared the growth of AP1 bacteria in normal and PCI-deficient plasma. As seen in Figure 5, bacterial proliferation is significantly accelerated when PCI has been removed from human plasma, suggesting that PCI is a relevant antimicrobial agent in human blood.

### PCI is associated with activated platelets

**Figure 6:**
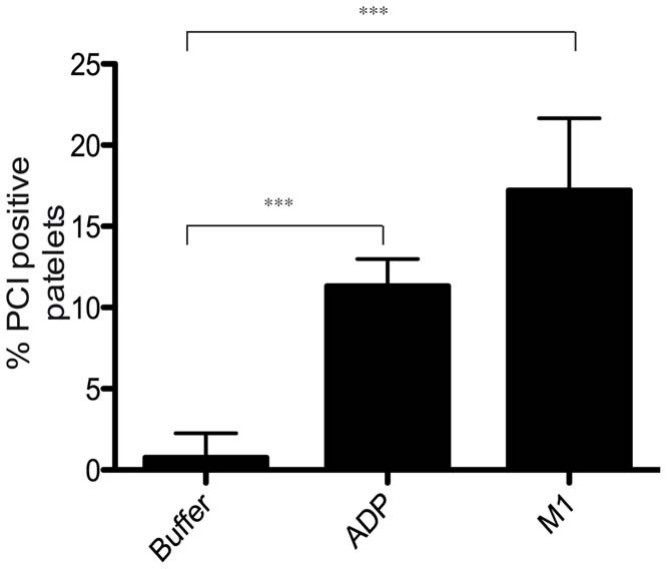
PCI associated with platelets. Platelet rich plasma was incubated with buffer, ADP (5 µM), or M1 protein (1 µg/ml). After 5 minutes, PCI was detected on the platelet surface using flow cytometry. The percentage of the platelet population that presented PCI on their surface was determined using Cell Quest Software (BD Biosciences). ADP and M1 protein stimulated PCI associated to platelets. Statistical significance was determined using students T test. ***  = P<0.001. Values are means, vertical lines show SEM; n = 4.

PCI is contained within the alpha-granules of platelets and may be released on activation [25]. It has previously been reported that M1 protein from *Streptococcus pyogenes* can stimulate platelet activation and that activated platelets are present at the site of streptococcal infection [26]. We therefore set about to determine whether PCI can be localized on platelets in response to the physiological activation (ADP) or bacterial activation (M1 protein). Unstimulated platelets had background levels of PCI on their surface. Following ADP activation, 11% of the platelet population had PCI on their surface and this was further increased to 17% on treatment with M1 protein (Figure 6). A proportion of PCI is likely to be released directly into the plasma, however our attempts to quantify platelet derived PCI in plasma failed due to technical limitations. We can therefore not differentiate between PCI released from platelets and PCI acquired from plasma and subsequently bound to platelets. These results do however demonstrate that PCI can be accumulated on the platelet surface during streptococcal infection and this may give rise to an increased PCI concentration at the local site of infection.

### PCI is accumulated at the infectious site in a patient with necrotizing fasciitis caused by *S. pyogenes*

**Figure 7:**
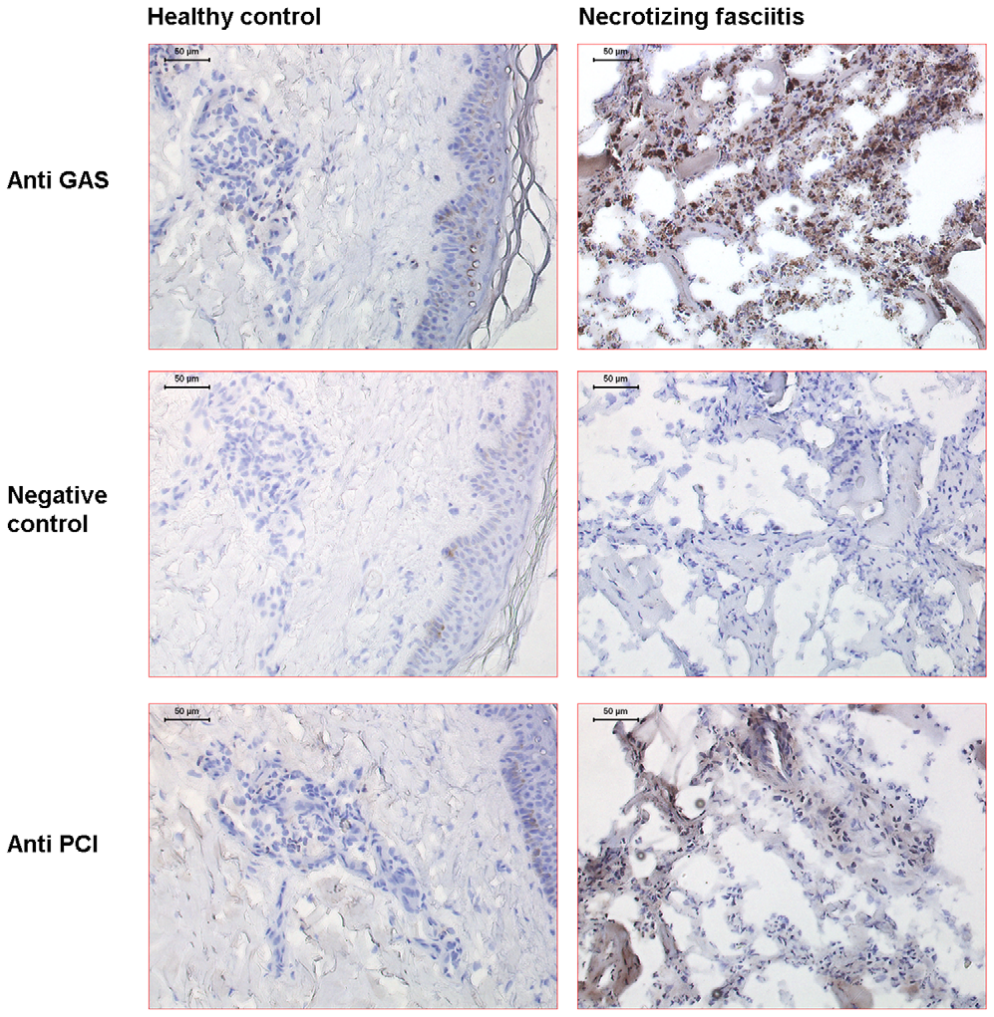
Immuno-histochemical analysis of tissue biopsies. Tissue biopsies were obtained at the epicenter of the infection from four patients with necrotizing fasciitis caused by *Streptococcus pyogenes*. For comparison, a skin biopsy was obtained from a healthy volunteer. The biopsies were sectioned and stained for *Streptococcus pyogenes* (GAS), PCI, and control staining where the primary antibody was omitted (ctrl). The figure shows 1 representative biopsy from a patient and the healthy control.

**Figure 8:**
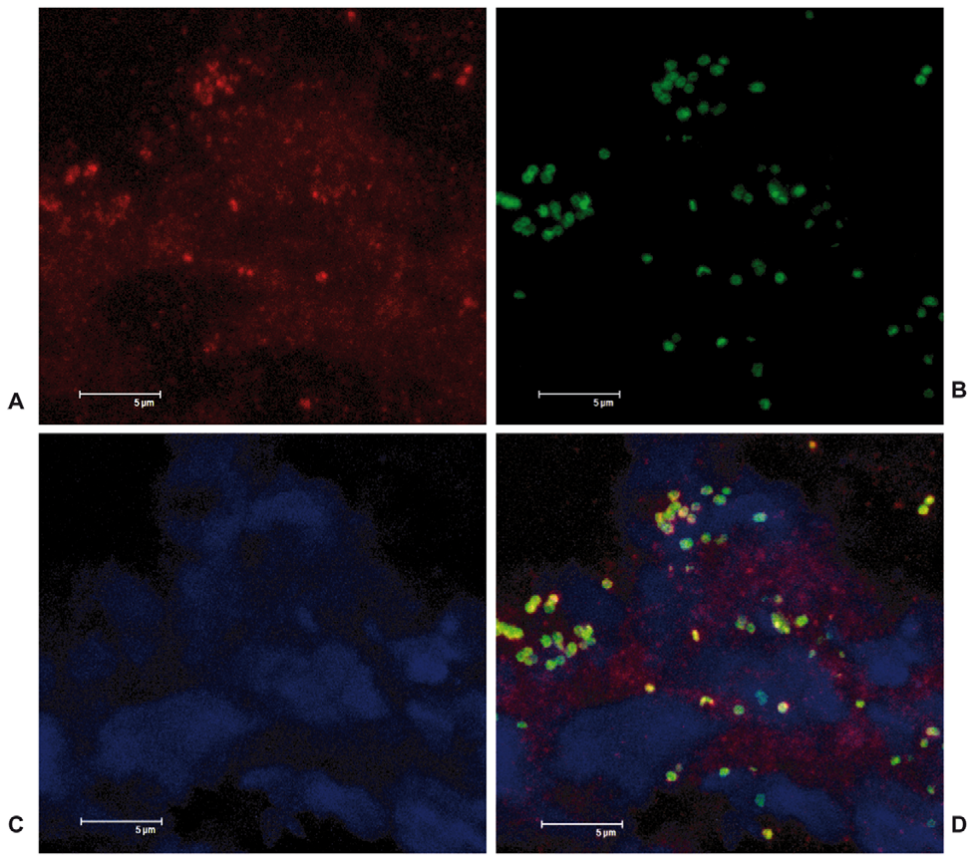
Co-localization of PCI and Streptococci at the infectious site. Tissue biopsies (see Figure 7) were stained for (Alexa 594, red, A) and *S. pyogenes* (Alexa 488, green, B). Cellular infiltrates are indicated in blue by the DNA binding stain DAPI (C). The yellow stain illustrates areas where bacteria and PCI are co-localized (D). Bar: 5 µm.

To test whether PCI levels increase at an infectious site, we analyzed biopsies derived from four patients with necrotizing fasciitis caused by *S. pyogenes* and a healthy control by immunohistochemistry. Bacterial colonization was demonstrated by employing an antibody against *S. pyogenes*, which demonstrates the presence of streptococci in the biopsy from the patient with necrotizing fasciitis, but not in the healthy control (Figure 7). When biopsies were immunostained for PCI, it was found that the protein was enriched at the infectious site, while it was not detectable in the control biopsy. PCI recruitment to the site of infection was localized in cellular infiltrates especially in areas with a lot of bacteria. To investigate these areas by confocal microscopy, biopsies were immunostained with antibodies against *S. pyogenes* and PCI. Once again the micrographs show that PCI and Streptococci are distributed throughout the cellular infiltrate (Figure 8A–C), and are most commonly co-localized (Figure 8D).

## Discussion

In the present study we show that PCI has a broad antibacterial activity towards many Gram negative and Gram positive microorganisms. Importantly, proteolytic processing of PCI is not required, since the protein is released into plasma in its antibacterial active form. This is in contrast to many other antimicrobial peptides/proteins that have to be generated from a precursor by the action of proteolytic active enzymes. Indeed, our data show that treatment of PCI with a panel of human proteinases including neutrophil-borne elastase, proteinases 3 or cathepsin G, does not diminish the antimicrobial activity of PCI. This feature has a major advantage, since due to neutrophil recruitment and their subsequent degranulation, neutrophil-derived proteinases are enriched at the infected site. Notably, especially in deep tissue infections such as necrotizing fasciitis with severe tissue degradation, extremely high proteolytic activity is recorded which is not only caused by host proteinases, but also by bacterial virulence factors (for a review see [27]). The increased protease activity at the site of infection may lead to an inactivation of antibacterial peptides that are not proteolytic resistant (for a review see [28]). This has been shown for instance for LL-37, which is probably cleaved by a streptococcal cysteine proteinase at the infected site in patients with severe tissue infections and completely broken down in wound fluids from patients with chronically infected venous ulcers [29],[30]. Our preliminary results with wound fluids from patients with chronically infected venous ulcers show that unlike LL-37, PCI is protected from degradation in this proteolytically potent environment (Malmström, Schmidtchen, and Herwald, unpublished results), suggesting that PCI is not only resistant to degradation by the host, but also by bacterial proteinases.

Analysis of biopsies from patients suffering from streptococcal necrotizing fasciitis reveal an accumulation of PCI at the infectious site and confocal microscopy studies show that most of the PCI is found co-localized with streptococci. These data confirm our *in vitro* and *ex vivo* results showing that PCI has an affinity for bacterial surfaces and they also allow the assumption that the protein exerts antibacterial activity at an infectious site. As PCI is found at low concentrations in plasma (4 to 6 µg/ml) an active transport of the proteins to or its up-regulation at the site of infection is required. Previous work has shown that platelets can infiltrate the infected site of patients suffering from soft tissue infections caused by *S. pyogenes* [26]. As the alpha-granules of platelets constitute a storage of PCI [25], we propose a model where PCI is released from platelets that are recruited to the site of infection. Unfortunately, we were not able to distinguish whether PCI was released from the α-granules or from the surface of platelets that have absorbed PCI from plasma. However, considering that platelets contain very little PCI (160 ng PCI/2×10^9^ cells) [25], it seems more likely that they absorbed it from plasma. At this point we cannot exclude that PCI synthesis is induced at the infectious site and therefore, in ongoing studies we address whether PCI generation can be triggered in different cell lines, including HepG2 cells, upon stimulation with inflammatory substances, such as IL-1β, IL-6, or TNFα.

The affinity of PCI for negatively charged lipids may help explain the mechanism by which the bacterial cell wall is disrupted. As visualized by negative staining electron microscopy, the interaction of PCI with bacteria leads to destruction of the bacterial cell wall and the release of cytosolic content, eventually leading to bacterial killing. Recently, Baumgärtner *et al.* reported that PCI is avidly internalized by human polymorph nuclear neutrophils (PMNs), and this involves phosphatidyl-ethanolamines [14]. The authors also reported that this interaction enhances the uptake of *E. coli* bacteria and, thus they suggest an important role for PCI in innate immunity [14]. Considering that the PCI-evoked release of bacterial cytosolic content will cause additionally inflammatory reactions at the infected site, it seems plausible that the host has developed a counteracting mechanism. Uptake of PCI-opsonized bacteria by PMNs before their destruction may lead to decreased inflammatory reactions, while still guaranteeing an efficient killing of the pathogen. Thus a synergistic effect of PCI resulting in bacterial recognition by PCI and their subsequent uptake by phagocytic cells followed by intracellular killing, is an attractive concept that would lead to a clearance of the infection and a dampening of inflammatory responses.

Taken together, our studies show a novel function for PCI as an antimicrobial agent against a broad arsenal of bacterial pathogens. This is mediated by the ability of PCI to interact with lipids leading to the efflux of bacterial cytosolic content. When analyzing tissue biopsies we find an accumulation of PCI at the infectious site. These findings suggest an important and novel role of PCI in innate immunity.

## Materials and Methods

### Ethics statement

The Human Subjects Review Committee of the University of Toronto and of Lund University approved the studies, and written, informed consent from the patients and volunteers was received.

### Reagents

Fresh frozen plasma from healthy individuals were obtained from the blood bank at Lund University Hospital, Lund, Sweden, and kept frozen at −80°C until use. Protein C Inhibitor deficient plasma was prepared as described [31]. M1 protein was purified from the supernatant of *S. pyogenes* MC25, as previously described [32].

### Antimicrobial peptides and protein

SEK20 (SEKTLRKWLKMFKKRQLELYL) and LL-37 (LLGDFFRKSK EKIGKEFKRI VQRIKDFLRN LVPRTES) were synthesized by Innovagen AB, Lund, Sweden. The purity (>95%) and molecular weight of these peptides was confirmed by mass spectral analysis (MALDI.TOF Voyager). Recombinant protein C inhibitor was purified as previously described [33].

### Microorganisms

*Escherichia coli 37.4* and ATCC25922, *Pseudomonas aeruginosa* ATCC27853, *Staphylococcus aureus* ATCC29213, *Bacillus subtilis* ATCC6633 bacterial isolates, and the fungal isolate *Candida albicans* ATCC90028 were grown as described elsewhere [34]. The *Streptococcus pyogenes* AP1 (40/58) strain of the M1 serotype was provided by the WHO (World Health Organization) Streptococcal Reference Laboratory in Prague, Czech Republic and cultured as previously described [35].

### Bactericidal assays

Radial diffusion assays were performed as described previously [36]. Briefly, bacteria were grown to mid-logarithmic phase in 10 ml full strength 3% (w/v) tryptic soy broth (TSB) (Becton Dickinson, Franklin Lakes, NJ, USA). The bacteria were washed once in 10 mM Tris, pH 7.4 and then 2×10^6^ CFU were added to 5 ml of the underlay agarose gel consisting of 0.03% (w/v) TSB, 1% (w/v) low-electroendosmosistype (Low-EEO) agarose (Sigma) and a final concentration of 0.02% (v/v) Tween-20. The underlay was poured into a Petri dish. After the agarose had solidified, 4 mm diameter wells were punched and 6 µl of test samples were added to each well. Samples were allowed to diffuse into the gel for 3 h in 37°C and then the underlay gel was covered with 5 ml overlay (6% TSB, 1% Low-EEO agarose). Antibacterial activity was visualized as a clear zone around each well after overnight incubation at 37°C of the plate. The activities of the peptides are presented as diameter of clear zone-well diameter.

### Hemolytic assay

EDTA-blood was centrifuged at 800 g for 10 min and plasma and buffy coat removed. Erythrocytes were washed three times and resuspended in 5% PBS, pH 7.4. The cells were incubated with end-over-end rotation for 1 h at 37°C in the presence of SEK20 or LL-37 (3–60 µM). 2% Triton X-100 (Sigma-Aldrich) served as positive control. The samples were then centrifuged at 800 g for 10 min. Hemoglobin release was measured as the absorbance at λ 540 nm and the values are expressed as % of TritonX-100 induced hemolysis.

### Liposome preparation and leakage assay

Dry lipid films were prepared by dissolving dioleoylphosphatidylcholine (Avanti Polar Lipids, Alabaster, AL) (30 mol%), dioloeolphosphatidic acid (30 mol %) and cholesterol (Sigma, St Louis, MO) (40 mol%) in chloroform, and removing the solvent by evaporation under vacuum overnight. Subsequently, buffer solution containing 10 mM Tris, pH 7.4, was added together with 0.1 M carboxyfluorescein (CF) (Sigma, St Louis, MO). After hydration, the lipid mixture was subjected to eight freeze-thaw cycles consisting of freezing in liquid nitrogen and heating to 60°C. Unilamellar liposomes with a diameter of about 140 nm (as found with cryo-TEM and dynamic light scattering; results not shown) were generated by multiple extrusions through polycarbonate filters (pore size 100 nm) mounted in a LipoFast miniextruder (Avestin, Ottawa, Canada). Untrapped carboxyfluorescein was then removed by filtration through two subsequent Sephadex G-50 columns with the relevant Tris buffer as eluent. Both extrusion and filtration was performed at 22°C. In the liposome leakage assay, the well known self-quenching of CF was used. Thus, at 100 mM CF is self-quenched, and the recorded fluorescence intensity from liposomes with entrapped CF is low. On leakage from the liposomes, released CF is dequenched, and hence fluoresces. The CF release was determined by monitoring the emitted fluorescence at 520 nm from a liposome dispersion (10 mM lipid in 10 mM Tris pH 7.4). An absolute leakage scale is obtained by disrupting the liposomes at the end of the experiment through addition of 0.8 mM Triton X100 (Sigma, St Louis, MO), thereby causing 100% release and dequenching of CF. A SPEX-fluorolog 1650 0.22-m double spectrometer (SPEX Industries, Edison, NJ) was used for the liposome leakage assay.

### Lactate dehydrogenase (LDH) assay

HaCaT keratinocytes were grown to confluence in 96 well plates (3000 cells/well) in DMEM, 10% FCS. The medium was removed and the cells were subsequently washed with 100 µl DMEM. 100 µl of SEK20 or LL-37 (0, 3, 6, 30, 60 µM) diluted in DMEM were added in triplicates. The LDH based TOX-7 kit (Sigma-Aldrich) was used to measure the viability of the cells.

### Viable count assay

Bacteria were cultivated overnight in Todd-Hewitt medium (TH; Difco) at 37°C. 250 µl were transferred to 10 ml TH-medium and grown to mid-log phase (A_620_≈0.4). The bacterial solution was washed 3 times in 10 mM Tris, 5 mM glucose, pH 7.4 (Tris-HCl buffer). 5 µl (2×10^6^ cfu/ml) was added to the respective antimicrobial substance at a total volume of 17 µl and incubated for 1 h at 37°C. After the incubation 500 µl Tris buffer was added to the mixture and 100 µl was transferred to Todd-Hewitt broth agar plate to determine the bacterial growth. Plates were incubated overnight at 37°C and the number of colony forming units (cfu) was determined.

### Electron microscopy

The antimicrobial effect of PCI against *E. coli* and *S. pyogenes* was analyzed by negative staining and electron microscopy as previously described [37]. Bacteria were diluted to a 1% solution with TBST (20 mM Tris, 150 mM NaCl, 0.05% Tween, pH 7.4) and 10 µl incubated with PCI at a concentration of 4 µM for 90 min at 37°C. 5 µl aliquots were adsorbed onto carbon-coated grids for 1 min, washed with two drops of water, and stained on two drops of 0.75% uranyl formate. The grids were rendered hydrophilic by glow discharge at low pressure in air. Specimens were observed in a Jeol JEM 1230 electron microscope operated at 60 kV accelerating voltage. Images were recorded with a Gatan Multiscan 791 CCD camera.

### Plasma absorption

Bacteria, grown to mid-exponential growth phase were washed and resuspended in PBST (PBS+0.05% Tween-20). 250 µl of the bacterial solution (2×10^9^ bacteria/ml) was incubated with 1.5 ml citrate plasma for 1 h at 37°C. The bacterial cells were collected, washed with PBST including 0.5 M NaCl and bound proteins eluted with 0.1 M glycine–HCl, pH 2.0. The pH of the eluted material was raised to 7.5 by addition of 1 M Tris. Precipitated material was dissolved in SDS sample buffer and subjected to Tricine-SDS gel electrophoresis and Western blot analysis.

### Western blot analysis

Samples were boiled for 5 min in an equal volume of sample buffer containing 2% SDS and 5% 2-mercaptoethanol and run on SDS-PAGE. Bio-rad kaleidoscope prestained standards were used as molecular weight markers. Separated proteins were transferred to polyvinylidene difluoride (PVDF) membranes (Amersham Biosciences). Membranes were blocked with PBST (PBS+0.05% Tween-20) containing 5% dry milk powder (blocking buffer) overnight at 4°C and then incubated with primary antibodies (rabbit anti-PCI K88032 1∶1000) in blocking buffer for 1 h at 37°C. After a washing step with PBST with 0.35 M NaCl, the membranes were incubated with HRP-conjugated secondary antibodies (goat anti-rabbit IgG 1∶10000) in blocking buffer for 1 h at 37°C. The membranes were washed and bound antibodies detected by chemiluminescence.

### Bacterial growth in PCI deficient plasma

*S. pyogenes* was cultivated in human plasma and PCI-deficient plasma at 37°C. Bacterial suspensions were diluted 10000 times in 10 mM Tris, containing 5 mM glucose (pH 7.4) and transferred to TH-agar plates at different time points (0, 4 and 8 h) to monitor bacterial growth. Plates were incubated overnight at 37°C and the number of bacteria determined.

### Platelet analyses using flow cytometry

Blood samples were collected from healthy donors who had not taken antiplatelet medication in the previous ten days. Five ml of blood was collected into citrated vacuum tubes. Platelet-rich plasma (PRP) was prepared by centrifugation at 150×g for 10 minutes. Twenty µl of PRP was incubated at room temperature with 25 µl of HEPES buffer pH 7.4, either in the presence or absence of 5 µM adenosine diphosphate (ADP) or M1 protein (1 µg/ml). After 5 minutes, primary antibodies were added (rabbit anti-PCI K88032 1∶100) and incubated for 5 minutes. Five µl of fluorochrome conjugated secondary antibody (anti Rabbit FITC) was then added and after 5 minutes the incubation was stopped by addition of 0.5% formaldehyde in ice cold PBS. Samples were analysed using a FACSCalibur flow cytometer in logarithmic mode with a gate setting on the platelet population. 50,000 cells were acquired and analysed using Cell Quest software (Becton Dickinson).

### Immunohistochemical staining of tissue biopsies

A snap-frozen tissue biopsy collected from patients with necrotizing fasciitis caused by group A streptococcus (GAS) was stained and compared with a snap-frozen punch biopsy taken from a healthy volunteer. The biopsies were cryostat-sectioned to 8 µm, fixed in 2% freshly prepared formaldehyde in PBS and stained. Tissue sections were initially blocked with 10% fetal calf serum in Earl's balanced salt solution (BSS) with 0.1% saponin for 30 minutes at room temperature, followed by additional blocking with 1% H_2_O_2_ in BSS-saponin and an avidin and biotin blocking (Vector laboratories). Primary antibodies were diluted in BSS solution containing saponin and 0.02% NaN_3_ and incubated over night at room temperature. *S. pyogenes* bacteria were identified with a polyclonal rabbit antiserum specific for the Lancefield group A carbohydrate (diluted 1∶10,000; Difco), while PCI was identified with a polyclonal rabbit antiserum (1 µg/ml) K88032. After incubation, tissue sections were washed and blocked with 1% normal goat serum in BSS-saponin before addition of biotinylated goat anti-rabbit IgG (diluted 1∶500, Vector laboratories) diluted in 1% normal goat serum in BSS-saponin. Avidin-peroxidase solution was added (Vectastain-Elite; Vector Laboratories) and the color reaction developed by the addition of 3,3-diaminobenzidine (Vector Laboratories) followed by counterstaining with hematoxylin. PCI and streptococci were also visualized with immunoflourescence stainings. The tissue was initially blocked with an avidin/biotin blocking kit (Vector laboratories) whereafter PCI was identified with a monoclonal mouse antibody (5 µg/ml) API-93 followed by a blocking step with 1% normal goat serum. This was followed by incubation with a biotinylated antibody against mouse IgG (diluted 1∶600, Dako), and subsequently with a streptavidin-conjugated fluorophor (Alexa 594, diluted 1∶500, Molecular Probes). After yet another round of blocking with avidin/biotin streptococci were detected using a biotinylated polyclonal rabbit antiserum specific for the Lancefield group A carbohydrate (16 µg/ml, Difco) followed by incubation with a second streptavidin-conjugated fluorophore (Alexa 488, diluted 1∶600, Dako). All antibodies and fluorochromes were diluted in PBS-saponin-BSA-c, while washes were done with PBS-saponin. The immunofluorescence stainings were evaluated by a Leica confocal scanner TCP SP II coupled to a Leica DMR microscope.

### Statistical analysis

Statistical analysis was performed using GraphPadPrism 4.00. For viable count data the p value was determined using a one sample T test with a theoretical mean set as 100% survival based on untreated samples. For flow cytometry data the p value was determined using students T test.

## Supporting Information

Figure S1 Antimicrobial effect of LL-37, SEK20, and GKD25 on *E. coli* and *S. pyogenes. E. coli* 37.4 (A) and *S. pyogenes* strain AP1 (B) were incubated with LL-37 (4 µM), SEK20 (4 µM), GKD25 (4 µM), or left untreated. GDK25 is a peptide derived from high molecular weight kininogen which has been shown to lack antimicrobial activity [16]. To quantify bactericidal activity reaction mixtures were plated on TH broth agar followed by incubation over night at 37°C and the number of cfus was determined. *E. coli* (C and D) and AP1 (E and F) bacteria before (C and E) and after (D and F) treatment with GKD25 (2 µM) were subjected to negative staining electron microscopy (bar = 1 µm). The figure shows that LL-37 and SEK20 possess antimicrobial activity, but not GDG25.
(6.08 MB TIF)

Figure S2 Antimicrobial effect of protease-treated PCI on *E. coli* and *S. pyogenes*. PCI (100 nM, final concentration) was incubated with human activated protein C (aPC), factor Xa (FXa), plasma kallikrein (PKa), thrombin, elastase, cathepsin G (cath. G), proteinase 3 (prot. 3) or left untreated (PCI) for 30 min at 37°C. All proteinases were used at a final concentration of 10 nM. PCI and proteinases-treated PCI was incubated with *E. coli* and *S. pyogenes* bacteria as described and Materials and Methods followed by negative staining electron microscopy analysis. Untreated bacteria were used as negative control (A). The figure shows that proteinase-treatment did not impair the antimicrobial activity of PCI. The percentage killing judged from the electron microscopic analysis is indicated in each micropgraph.
(8.73 MB TIF)

Figure S3 Antimicrobial effect of PCI (100 nM) on *E. coli* and *S. pyogenes*. *E. coli* (A and C) and *S. pyogenes* strain AP1 (B and D) were incubated PCI (C and D) at a concentration of 100 nM, or left untreated (A and B) and analyzed by negative electron microcopy. Scale bar = 200 nm).
(6.28 MB TIF)

## References

[1] Areschoug T, Gordon S (2008). Pattern recognition receptors and their role in innate immunity: focus on microbial protein ligands.. Contrib Microbiol.

[2] Beutler BA (2009). TLRs and innate immunity.. Blood.

[3] Taylor PR, Martinez-Pomares L, Stacey M, Lin HH, Brown GD (2005). Macrophage receptors and immune recognition.. Annu Rev Immunol.

[4] Nordahl EA, Rydengard V, Nyberg P, Nitsche DP, Morgelin M (2004). Activation of the complement system generates antibacterial peptides.. Proc Natl Acad Sci U S A.

[5] Eliasson M, Egesten A (2008). Antibacterial chemokines–actors in both innate and adaptive immunity.. Contrib Microbiol.

[6] El Karim IA, Linden GJ, Orr DF, Lundy FT (2008). Antimicrobial activity of neuropeptides against a range of micro-organisms from skin, oral, respiratory and gastrointestinal tract sites.. J Neuroimmunol.

[7] Seebah S, Suresh A, Zhuo S, Choong YH, Chua H (2007). Defensins knowledgebase: a manually curated database and information source focused on the defensins family of antimicrobial peptides.. Nucleic Acids Res.

[8] Sorensen OE, Follin P, Johnsen AH, Calafat J, Tjabringa GS (2001). Human cathelicidin, hCAP-18, is processed to the antimicrobial peptide LL-37 by extracellular cleavage with proteinase 3.. Blood.

[9] Schultz H, Weiss JP (2007). The bactericidal/permeability-increasing protein (BPI) in infection and inflammatory disease.. Clin Chim Acta.

[10] Soehnlein O, Lindbom L (2009). Neutrophil-derived azurocidin alarms the immune system.. JLeukoc Biol.

[11] Rydengard V, Olsson AK, Mörgelin M, Schmidtchen A (2007). Histidine-rich glycoprotein exerts antibacterial activity.. FEBS J.

[12] Pratt CW, Church FC (1992). Heparin binding to protein C inhibitor.. J Biol Chem.

[13] Suzuki K (2008). The multi-functional serpin, protein C inhibitor: beyond thrombosis and hemostasis.. J Thromb Haemost.

[14] Baumgärtner P, Geiger M, Zieseniss S, Malleier J, Huntington JA (2007). Phosphatidylethanolamine critically supports internalization of cell-penetrating protein C inhibitor.. J Cell Biol.

[15] Andersson E, Rydengård V, Sonesson A, Mörgelin M, Björck L (2004). Antimicrobial activities of heparin-binding peptides.. Eur J Biochem.

[16] Frick IM, Åkesson P, Herwald H, Mörgelin M, Malmsten M (2006). The contact system–a novel branch of innate immunity generating antibacterial peptides.. Embo J.

[17] Bevins CL (2003). Antimicrobial peptides as effector molecules of mammalian host defense.. Contrib Microbiol.

[18] Nordahl EA, Rydengård V, Nyberg P, Nitsche DP, Mörgelin M (2004). Activation of the complement system generates antibacterial peptides.. Proc Natl Acad Sci USA.

[19] Sørensen OE, Follin P, Johnsen AH, Calafat J, Tjabringa GS (2001). Human cathelicidin, hCAP-18, is processed to the antimicrobial peptide LL-37 by extracellular cleavage with proteinase 3.. Blood.

[20] Rydengård V, Olsson AK, Mörgelin M, Schmidtchen A (2007). Histidine-rich glycoprotein exerts antibacterial activity.. Febs J.

[21] Pohl J, Pereira HA, Martin NM, Spitznagel JK (1990). Amino acid sequence of CAP37, a human neutrophil granule-derived antibacterial and monocyte-specific chemotactic glycoprotein structurally similar to neutrophil elastase.. FEBS Lett.

[22] Li W, Adams TE, Kjellberg M, Stenflo J, Huntington JA (2007). Structure of native protein C inhibitor provides insight into its multiple functions.. J Biol Chem.

[23] Lundqvist K, Herwald H, Sonesson A, Schmidtchen A (2004). Heparin binding protein is increased in chronic leg ulcer fluid and released from granulocytes by secreted products of *Pseudomonas aeruginosa*.. Thromb Haemost.

[24] Geiger M (2007). Protein C inhibitor, a serpin with functions in- and outside vascular biology.. Thromb Haemost.

[25] Prendes MJ, Bielek E, Zechmeister-Machhart M, Vanyek-Zavadil E, Carroll VA (1999). Synthesis and ultrastructural localization of protein C inhibitor in human platelets and megakaryocytes.. Blood.

[26] Shannon O, Hertzen E, Norrby-Teglund A, Mörgelin M, Sjöbring U (2007). Severe streptococcal infection is associated with M protein-induced platelet activation and thrombus formation.. Mol Microbiol.

[27] Olsen RJ, Musser JM (2009). Molecular Pathogenesis of Necrotizing Fasciitis.. Annu Rev Pathol: in press.

[28] Nizet V (2006). Antimicrobial peptide resistance mechanisms of human bacterial pathogens.. Curr Issues Mol Biol.

[29] Johansson L, Thulin P, Sendi P, Hertzén E, Linder A (2008). Cathelicidin LL-37 in severe *Streptococcus pyogenes* soft tissue infections in humans.. Infect Immun.

[30] Schmidtchen A, Frick IM, Andersson E, Tapper H, Björck L (2002). Proteinases of common pathogenic bacteria degrade and inactivate the antibacterial peptide LL-37.. Mol Microbiol.

[31] Elisen MG, von dem Borne PA, Bouma BN, Meijers JC (1998). Protein C inhibitor acts as a procoagulant by inhibiting the thrombomodulin-induced activation of protein C in human plasma.. Blood.

[32] Collin M, Olsen A (2000). Generation of a mature streptococcal cysteine proteinase is dependent on cell wall-anchored M1 protein.. Mol Microbiol.

[33] Elisen MG, Maseland MH, Church FC, Bouma BN, Meijers JC (1996). Role of the A+ helix in heparin binding to protein C inhibitor.. Thromb Haemost.

[34] Malmsten M, Davoudi M, Walse B, Rydengård V, Pasupuleti M (2007). Antimicrobial peptides derived from growth factors.. Growth Factors.

[35] Åkesson P, Cooney J, Kishimoto F, Björck L (1990). Protein H–a novel IgG binding bacterial protein.. Mol Immunol.

[36] Lehrer RI, Rosenman M, Harwig SS, Jackson R, Eisenhauer P (1991). Ultrasensitive assays for endogenous antimicrobial polypeptides.. J Immunol Methods.

[37] Bengtson SH, Sandén C, Mörgelin M, Marx PF, Leeb-Lundberg FLM (2009). Activation of TAFI on the surface of *Streptococcus pyogenes* evokes inflammatory reactions by modulating the kallikrein/kinin system.. J Innate Immun.

