# Supplementary figures and images for: Protein C Inhibitor—A Novel Antimicrobial Agent
Source: PLoS Pathog. 2009 Dec 18;5(12):e1000698. doi: 10.1371/journal.ppat.1000698 (PMC2788422; doi:10.1371/journal.ppat.1000698)

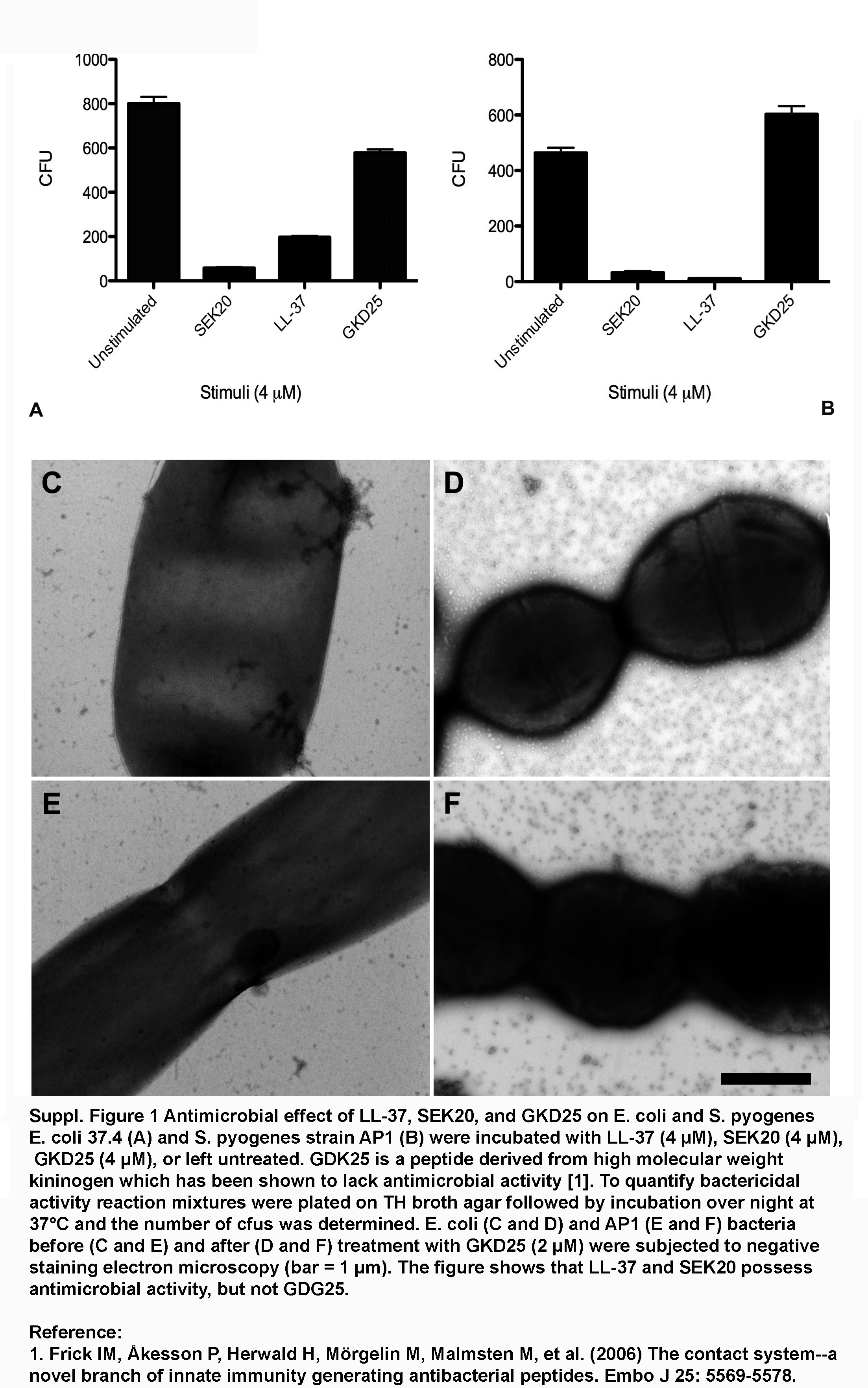

Supplement: Figure S1 — Antimicrobial effect of LL-37, SEK20, and GKD25 on E. coli and S. pyogenes. E. coli 37.4 (A) and S. pyogenes strain AP1 (B) were incubated with LL-37 (4 µM), SEK20 (4 µM), GKD25 (4 µM), or left untreated. GDK25 is a peptide derived from high molecular weight kininogen which has been shown to lack antimicrobial activity [16]. To quantify bactericidal activity reaction mixtures were plated on TH broth agar followed by incubation over night at 37°C and the number of cfus was determined. E. coli (C and D) and AP1 (E and F) bacteria before (C and E) and after (D and F) treatment with GKD25 (2 µM) were subjected to negative staining electron microscopy (bar = 1 µm). The figure shows that LL-37 and SEK20 possess antimicrobial activity, but not GDG25. (6.08 MB TIF) [file ppat.1000698.s001.tif]

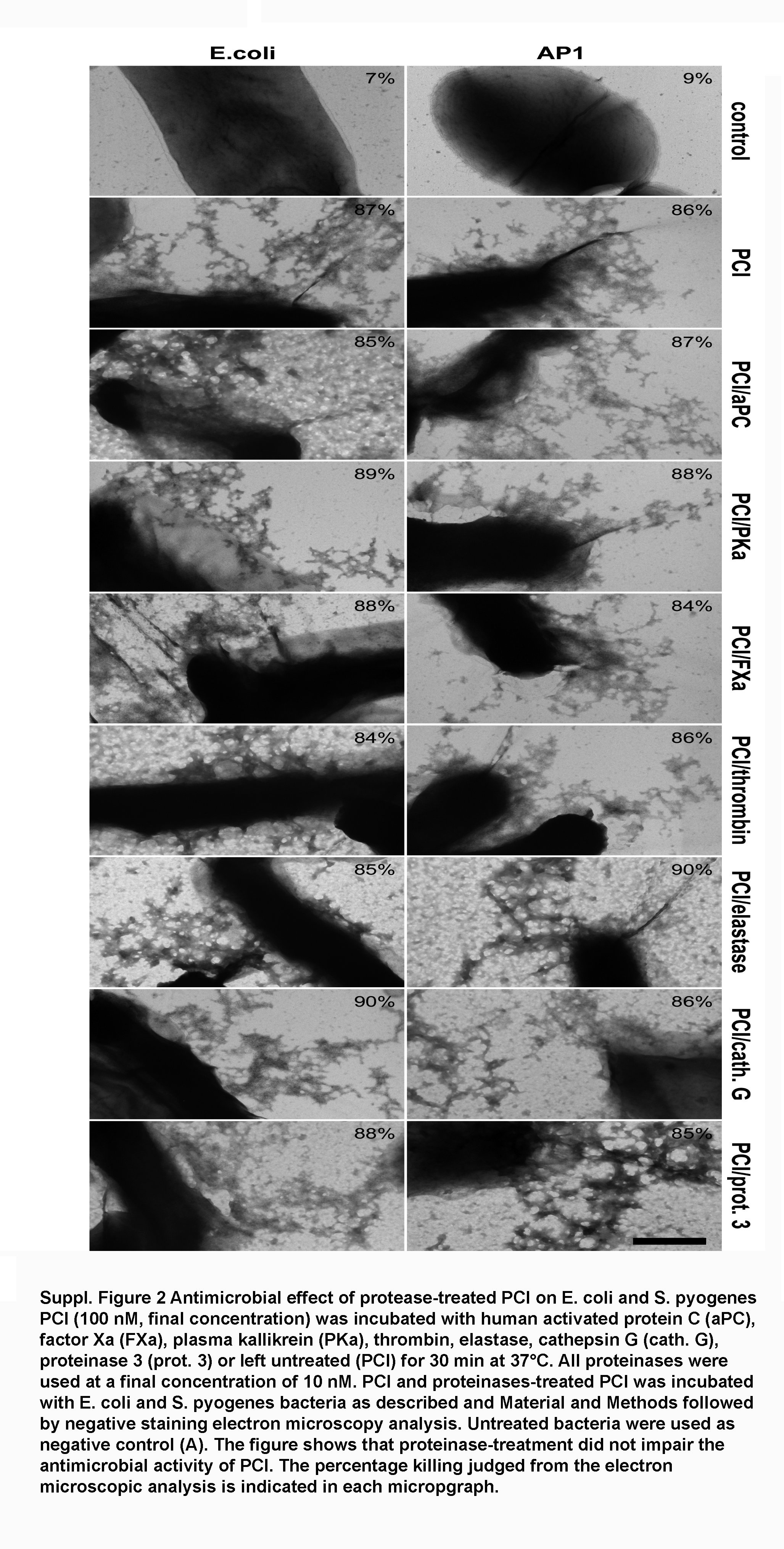

Supplement: Figure S2 — Antimicrobial effect of protease-treated PCI on E. coli and S. pyogenes. PCI (100 nM, final concentration) was incubated with human activated protein C (aPC), factor Xa (FXa), plasma kallikrein (PKa), thrombin, elastase, cathepsin G (cath. G), proteinase 3 (prot. 3) or left untreated (PCI) for 30 min at 37°C. All proteinases were used at a final concentration of 10 nM. PCI and proteinases-treated PCI was incubated with E. coli and S. pyogenes bacteria as described and Materials and Methods followed by negative staining electron microscopy analysis. Untreated bacteria were used as negative control (A). The figure shows that proteinase-treatment did not impair the antimicrobial activity of PCI. The percentage killing judged from the electron microscopic analysis is indicated in each micropgraph. (8.73 MB TIF) [file ppat.1000698.s002.tif]

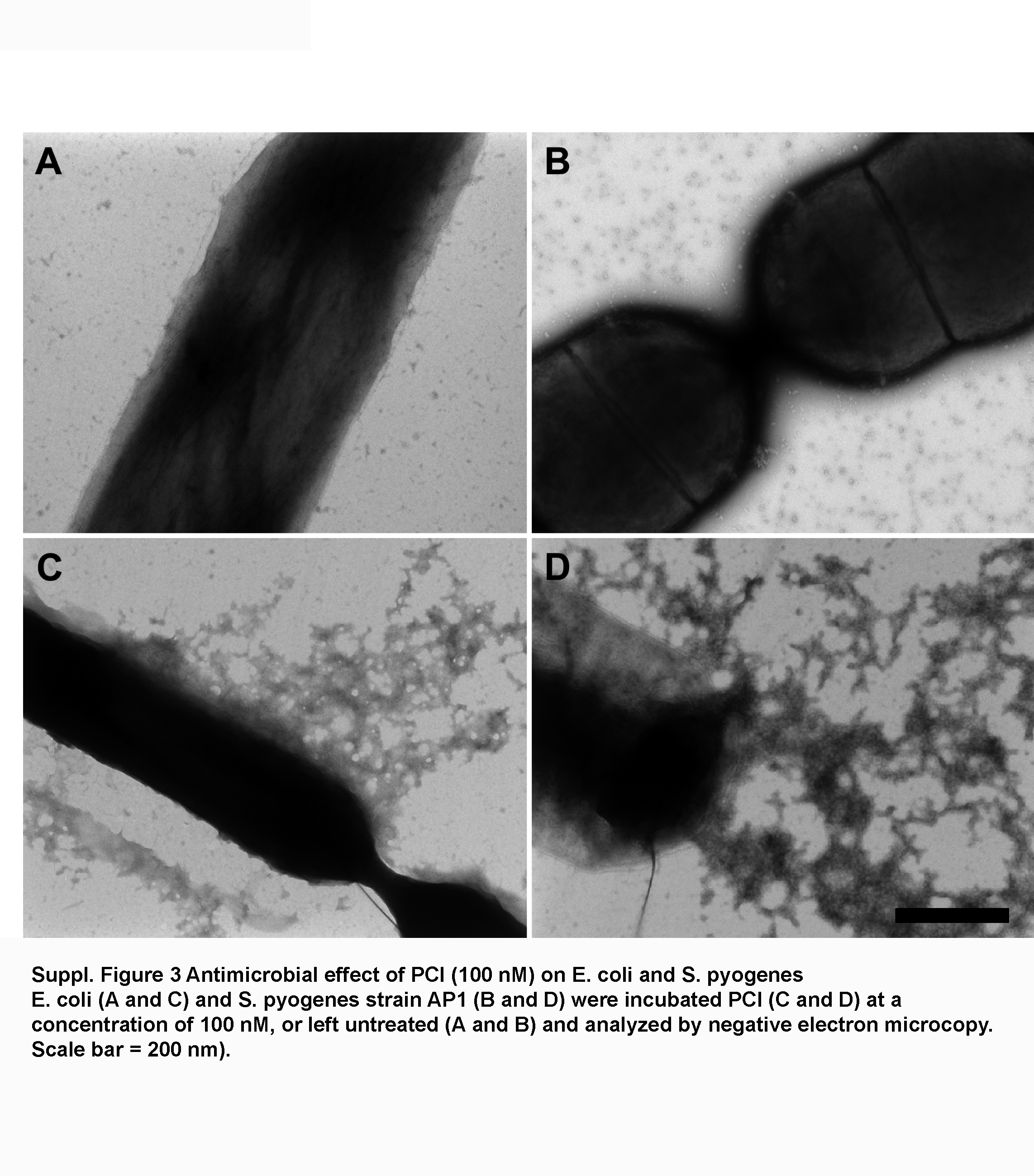

Supplement: Figure S3 — Antimicrobial effect of PCI (100 nM) on E. coli and S. pyogenes. E. coli (A and C) and S. pyogenes strain AP1 (B and D) were incubated PCI (C and D) at a concentration of 100 nM, or left untreated (A and B) and analyzed by negative electron microcopy. Scale bar = 200 nm). (6.28 MB TIF) [file ppat.1000698.s003.tif]
